# Supplementary material for: A Prospective Observational Cohort Study Comparing High-Complexity Against Conventional Pelvic Exenteration Surgery
Source: Cancers (Basel). 2025 Jan 1;17(1):111. doi: 10.3390/cancers17010111 (PMC11719841; doi:10.3390/cancers17010111)
Supplement: Supplementary file 1 [file cancers-17-00111-s001.zip › Supplementary File S1 - Outcome Definitions.pdf]

Outcome definitions:

- Overall survival – Kaplan-Meier open-ended time between surgery and death, with median and percentage survival at 5 years taken from the Kaplan-Meier curve.
- Disease-free survival – Kaplan-Meier open-ended time until any cancer recurrence appears, with median and percentage disease-free survival at 5 years taken from the Kaplan-Meier curve.
- Local-disease-free survival – Kaplan-Meier open-ended time until a local recurrence of cancer appears within the greater or lesser pelvis following PE, with median and percentage local-disease-free survival at 5 years taken from Kaplan-Meier curve.
- Perioperative mortality – death within 90 days from the date of surgery
- R0-resection – at least 1mm margin from any malignant cells be they continuous with the main tumour, or viable discontinuous tumour cells, as per contemporary UK Royal College of Pathology Guidelines for colorectal cancer, but adapted to other tumour types. Note that pelvic exenteration and hyperthermic intraperitoneal chemotherapy for disseminated peritoneal disease, or benign disease were excluded from this analysis.
    - Continuous R1-resection – where the R1 margin is continuous with the main tumour.
    - Discontinuous R1-resection – where the R1 margin is not continuous with the main tumour, constituting tumour fragmentation, tumour deposits, or malignant encapsulated or non-encapsulated lymph nodes.
- Index admission major morbidity – a Clavien-Dindo complication ≥3a during the admission immediately following pelvic exenteration
- Overall major morbidity – index admission major morbidity with the addition of any Clavien-Dindo complication ≥3a attributable to pelvic exenteration occurring following discharge, until the last date of follow-up or death. This did not include complications arising due to disease recurrence.
- Resource use – as per Unit Costs given in Table S1.
- Health-Related Quality of Life – EQ5D-5L, and Decision Regret Scores at pre-surgery baseline, 3 months, 6 months, and 12 months following PE.
- Median follow-up time – months between date of surgery and either date of death, or date of last known clinical or radiological follow-up.
