# Supplementary material for: A Prospective Observational Cohort Study Comparing High-Complexity Against Conventional Pelvic Exenteration Surgery
Source: Cancers (Basel). 2025 Jan 1;17(1):111. doi: 10.3390/cancers17010111 (PMC11719841; doi:10.3390/cancers17010111)
Supplement: Supplementary file 1 [file cancers-17-00111-s001.zip › Supplementary File S2 - Markov model parameters and assumptions.pdf]

Key parameters:	Justification
Two states (alive and dead)	Simplify Markov model, allows comparison with data taken from Koh et al [17]
3-month cycles	Frequency of EQ5D-5L data collection displayed in Figure 2 therefore maximising precision for this data
15-year time horizon	Kaplan-Meier survival curve implies benefit from pelvic exenteration lasts over at least 15 years
Discounting	Quality-adjusted life-years (QALYs) and costs discounted at 3.5 per year as per NICE reference case
Sensitivity analysis	See Table S4

	**No exenteration group [17]**	**Notes and assumptions**	**High-complexity pelvic exenteration**	**Notes and assumptions**	**Conventional pelvic exenteration**	**Notes and assumptions**
**Transition probabilities**						
Probability of death per cycle	0.0916	Calculated 3-monthly probability of death using no-surgery study population and assumed that this will probability will not change beyond 26.2 months in patients with active pelvic cancers	3-monthly probabilities from Weibull curve	Kaplan-Meier survival curve in Figure 1, converted into a parametric Weibull curve to predict 3-monthly chance of death over the 15-year time horizon.	3-monthly probabilities from Weibull curve	As per high-complexity group

Costs						
Mean inpatient costs to 3-months post-surgery (£)	£7,242	Mean costs taken from Figure 2 - approximately AUD 10,000<br><br>Converted from 2016 AUD to 2023 GBP (average exchange rate in 2016 was 0.5514)<br>Then converted using Bank of England inflation calculator*	£46,426	Mean perioperative costs = £45,733 (from Table 3)<br><br>+ any costs from discharge to 3-months post-surgery, note if patients did not have 3-months of follow up they were censored from this calculation: n = 243, mean £693)	£37,598	All below derived in same method as high-complexity,<br><br>Mean perioperative costs = £37,271 (from Table 3)<br><br>+ 0-3 month costs, n = 63, mean £327
Mean follow up costs 3 - 6 months (£)	£7,242	As above - approximately AUD 10,000	£1,034	3-6 month post-surgery costs for only patients with 6 months follow up: n = 221, mean = £573<br>+<br>Costs of any adjuvant chemotherapy or radiotherapy: n = 250, mean = £461	£1,455	3-6 month post-surgery costs, n = 60, mean = £570<br>+<br>Adjuvant treatment costs, n = 62, mean £885
Mean follow up costs 6 - 9 months (£)	£6,047	As above - approximately AUD 8,350	£170	Mean 6-9 month post-surgery costs for only patients with 9 months follow up, n = 215	£10	n = 58
Mean follow up costs 9 - 12 months (£)	£6,047	As above - approximately AUD 8,350	£174	Mean 9-12 month post-surgery costs for only patients with 12 months follow up, n = 194	£31	n = 56

Mean follow up costs 12 - 15 months (£)	£5,251	As above - approximately AUD 7,250	£61	Mean 12-15 month post-surgery costs for only patients with 15 months follow up, n = 178	£71	n = 52
Mean follow up costs 15 - 18 months (£)	£5,251	As above - approximately AUD 7,250	£117	Mean 15-18 month post-surgery costs for only patients with 18 months follow up, n = 163	£333	n = 50
Mean follow up costs 18 - 21 months (£)	£1,793	As above - approximately AUD 2,475	£21	Mean 18-21 month post-surgery costs for only patients with 21 months follow up, n = 154	£932	n = 46
Mean follow up costs 21 - 24 months (£)	£1,793	As above - approximately AUD 2,475	£152	Mean 21-24 month post-surgery costs for only patients with 24 months follow up, n = 143	£0	n = 43
Mean long-term costs per quarter (£)	£325	Then assumed that patients with active pelvic cancers will need a similar level of resources until they die	£151	Surveillance costs estimated at an annual CT chest/abdomen/pelvis and an annual MRI pelvis, with a colonoscopy every five years, this is divided by four to give cost per cycle, costs are added from second cycle	£151	As per high-complexity PE
**Utility Scores**						
First 3-month cycle	0.57698	From Table 1 divided QALYs by life years to get mean utility score over study period	0.695	EQ5D-3L utility scores derived from 5L utility scores as per NICE [35], mean utility score at 0-months and 3-months for each patient	0.765	As per high-complexity PE

				If EQ5D missing for 3, 6, and 12-months then patient removed, if only 3 or 6 months missing then assumed these scores mean of bracketing scores, if did not die and lost to follow up assumed EQ5D scores remained constant		
Second 3-month cycle	0.57698	As above	0.65	As above but mean between 3-month and 6-month EQ5D-3L utility scores	0.751	As per high-complexity PE
Third 3-month cycle	0.57698	As above	0.676	As above but mean between 6-month and 12-month EQ5D-3L utility scores,<br>as scores not taken at 9-months	0.783	As per high-complexity PE
Fourth 3-month cycle	0.57698	As above, continued to 9th cycle as this corresponds to the 26.2 months of available data in Koh et al 2016	0.676	Same score as per third 3-month cycle	0.783	As per high-complexity PE
Subsequent yearly decline	As per McNamara et al 2023 [65]	Assume declines at the same rate as the general population from baseline from 10th cycle on, assuming 44% female with median age of 60	As per McNamara et al 2023 [65]	Assume declines at the same rate as the general population from the reduced<br>baseline, assuming 52% female and starting mean age of 61	As per McNamara et al 2023 [65]	As per high-complexity PE, with 52% female and starting mean age of 61

				This trend is extrapolated from the conclusion that HrQoL remains stable from its level from one-year post-PE [66]		

GBP = Great British Pound, AUD = Australian Dollar, CT = computed tomography scan, MRI = magnetic resonance imaging scan, QALYs = quality-adjusted life-year, PE = pelvic exenteration

*Note that the same conversion approach was used to convert all currencies described in the introduction and discussion of the paper to 2023 GBP (£).

Other assumptions and limitations:

- Pre-hospital costing data for the conventional PE and high-complexity PE groups was not available for microcosting, and Koh et al 2016 found no significant differences in pre-hospital costs for no-PE vs PE, so excluded pre-hospital costing from the analysis [17].
- Post-discharge costs:
    - Post-discharge data for 7 high-complexity and 1 conventional PE cases lacked sufficient detail for bottom-up costing and were censored from post-discharge costing analysis.
    - For partially completed post-discharge data, reasonable estimates were made in consultation with clinical experts.
    - To maximise estimate accuracy, only patients with complete data covering a full 3-month Markov cycle were included, if they were lost to follow-up, deceased, or had follow-up <2 years they were censored from the costing for corresponding post-discharge Markov cycle.
    - Post-discharge costs included estimated surveillance costs, adjuvant treatment costs, and costs related to admissions, re-interventions, or imaging due to complications from surgery; costs for investigations or treatments related to recurrences were not included.
- Adjuvant treatment costs:
    - Data on whether adjuvant treatments were definitively given were unavailable for 5 high-complexity and 2 conventional PE cases, these were excluded from adjuvant treatment costing.

    - Chemotherapy doses were estimated based on a standard body surface area of 2 m^2 and a weight of 70 kg. If adjuvant chemotherapy cycle data was missing, patients were assumed to have completed the recommended course per University Hospital Southampton guidelines.
- Surveillance costs were estimated based on typical costs for rectal cancer patients.
