# Supplementary material for: A Prospective Observational Cohort Study Comparing High-Complexity Against Conventional Pelvic Exenteration Surgery
Source: Cancers (Basel). 2025 Jan 1;17(1):111. doi: 10.3390/cancers17010111 (PMC11719841; doi:10.3390/cancers17010111)
Supplement: Supplementary file 1 [file cancers-17-00111-s001.zip › Supplementary File S3 - Reporting checklists.pdf]

STROBE Statement—checklist of items that should be included in reports of observational studies

	Item	Recommendation	Location in subheading
**Title and abstract**	1	(*a*) Indicate the study's design with a commonly used term in the title or the abstract	Abstract
		(*b*) Provide in the abstract an informative and balanced summary of what was done and what was found	Abstract
**Introduction**			
Background/rationale	2	Explain the scientific background and rationale for the investigation being reported	Introduction as a whole
Objectives	3	State specific objectives, including any prespecified hypotheses	Paragraph 6
**Materials and Methods**			
Study design	4	Present key elements of study design early in the paper	Paragraph 1
Setting	5	Describe the setting, locations, and relevant dates, including periods of recruitment, exposure, follow-up, and data collection	Paragraph 1
Participants	6	(*a*) *Cohort study*—Give the eligibility criteria, and the sources and methods of selection of participants. Describe methods of follow-up	Paragraphs 1 and 2
		(*b*) *Cohort study*—For matched studies, give matching criteria and number of exposed and unexposed	Paragraph 2
Variables	7	Clearly define all outcomes, exposures, predictors, potential confounders, and effect modifiers. Give diagnostic criteria, if applicable	Paragraph 3 and Supplementary File 1
Data sources/ measurement	8*	For each variable of interest, give sources of data and details of methods of assessment (measurement). Describe comparability of assessment methods if there is more than one group	Paragraphs 1, 2, and 3
Bias	9	Describe any efforts to address potential sources of bias	Paragraph 1
Study size	10	Explain how the study size was arrived at	Paragraph 2
Quantitative variables	11	Explain how quantitative variables were handled in the analyses. If applicable, describe which groupings were chosen and why	Paragraphs 3, 4, 5, and 6

Statistical methods	12	(*a*) Describe all statistical methods, including those used to control for confounding	Paragraphs 4 and 6
		(*b*) Describe any methods used to examine subgroups and interactions	Paragraph 6
		(*c*) Explain how missing data were addressed	Supplementary File 2, Paragraph 1, 3 and 4
		(*d*) *Cohort study*—If applicable, explain how loss to follow-up was addressed	Paragraph 1 and 4
		(*e*) Describe any sensitivity analyses	Paragraph 6
**Results**			
Participants	13*	(a) Report numbers of individuals at each stage of study—eg numbers potentially eligible, examined for eligibility, confirmed eligible, included in the study, completing follow-up, and analysed	Paragraph 1
		(b) Give reasons for non-participation at each stage	Paragraph 1
		(c) Consider use of a flow diagram	Graphical abstract
Descriptive data	14*	(a) Give characteristics of study participants (eg demographic, clinical, social) and information on exposures and potential confounders	Table 1 and paragraph 1
		(b) Indicate number of participants with missing data for each variable of interest	Table 1, Table 3, Table S2, Table S3, and Supplementary File 2
		(c) *Cohort study*—Summarise follow-up time (eg, average and total amount)	Figure 1, Table 2 and paragraph 3
Outcome data	15*	*Cohort study*—Report numbers of outcome events or summary measures over time	Table 2, Table 3, Figure 1, Figure 2, paragraphs 2 – 5
Main results	16	(*a*) Give unadjusted estimates and, if applicable, confounder-adjusted estimates and their precision (eg, 95% confidence interval). Make clear which confounders were adjusted for and why they were included	Throughout results
		(*b*) Report category boundaries when continuous variables were categorized	N/A
		(*c*) If relevant, consider translating estimates of relative risk into absolute risk for a meaningful time period	N/A
Other analyses	17	Report other analyses done—eg analyses of subgroups and interactions, and sensitivity analyses	Throughout results
**Discussion**			
Key results	18	Summarise key results with reference to study objectives	Paragraph 1

Limitations	19	Discuss limitations of the study, taking into account sources of potential bias or imprecision. Discuss both direction and magnitude of any potential bias	Limitations section – from paragraph 9 – 12 of discussion
Interpretation	20	Give a cautious overall interpretation of results considering objectives, limitations, multiplicity of analyses, results from similar studies, and other relevant evidence	Throughout discussion
Generalisability	21	Discuss the generalisability (external validity) of the study results	Conclusions section – paragraph 14 of discussion
**Other information**			
Funding	22	Give the source of funding and the role of the funders for the present study and, if applicable, for the original study on which the present article is based	Funding section

Note: Adapted from www.strobe-statement.org with references to case-control and cross-sectional studies removed.

Section/Topic	CONSORT-PRO Item	Recommended Content	Area addressed
**Title and Abstract**			
	P1b	The PRO should be identified in the abstract as a primary or secondary outcome.	Abstract
**Introduction**			
Background and objectives	2a	The scientific background and explanation of rationale of PRO assessment should be included.	Introduction – paragraph 4
	P2b	The PRO hypothesis should be stated, and relevant domains identified, if applicable.	Introduction – paragraph 6
**Methods**			
Participants	4a	PRO-specific criteria are required only if PROs were used for eligibility or stratification.	N/A
Outcomes	P6a	Evidence of PRO instrument validity and reliability should be provided or cited if available including the person completing the PRO and methods of data collection (paper, telephone, electronic).	Materials and Methods – paragraph 3
Sample size	7a	Sample size determination is required only if PRO is a primary study outcome.	N/A
**Randomization**			
Statistical methods	P12a	Statistical approaches for dealing with missing data are explicitly stated.	Materials and Methods – paragraph 3 and 4
Results			
Participant flow	13a	The number of PRO outcome data at baseline and at subsequent time points should be transparent.	Results – Figure 2 and Table S2
Baseline data	15	PRO data in the table showing baseline demographic and clinical characteristics for each group should be included.	Results – Table 1
Numbers analyzed	16	For each group, the number of participants (denominator) included in each analysis and whether the analysis was by original assigned groups) is required for PRO results.	Table S2
Outcomes and estimation	17a	The estimated effect size and its precision such as 95% confidence interval should be presented for multidimensional PROs from each domain and time point.	Table S2
Ancillary analyses	18	Results of any other PRO analyses performed, including subgroup analyses and adjusted analyses, distinguishing pre-specified from exploratory should be presented, where relevant.	N/A

**Discussion**			
Limitation	P21/21	PRO-specific limitations and implications for generalizability and clinical practice should be presented.	Limitations – paragraph 11 of discussion
Interpretation	22	PRO data should be interpreted in relation to clinical outcomes including survival data, where relevant.	Discussion – paragraph 5

Calvert M, Blazeby J, Altman DG, et al. Reporting of patient-reported outcomes in randomized trials: the CONSORT PRO extension. JAMA. 2013;309(8):814-822. doi:10.1001/jama.2013.879 Note: The CONSORT-PRO Extension should be used with the CONSORT 2010 Statement and any other relevant CONSORT Extensions, found at consort-statement.org
