# Supplementary material for: A Prospective Observational Cohort Study Comparing High-Complexity Against Conventional Pelvic Exenteration Surgery
Source: Cancers (Basel). 2025 Jan 1;17(1):111. doi: 10.3390/cancers17010111 (PMC11719841; doi:10.3390/cancers17010111)
Supplement: Supplementary file 1 [file cancers-17-00111-s001.zip › Table S1 - Unit Costs.pdf]

Item	Unit	Cost Assigned (£)	Source of cost
**Operation costs**			
Permacol porcine dermis 18x28cm	Patient	5760	NHS supply chain FVQ3394
Surgimend mesh 4.0mm 10x15cm	Patient	2299	Q Medical
Surgimend mesh 4.0mm 20x30cm	Patient	8,572	Q Medical
Strattice extra thick 10x16cm	Patient	2330.01	NHS supply chain FXS332
Strattice extra thick 20x25cm	Patient	7,999.33	NHS supply chain FXS358
Cook Biodesign mesh - 13x15cm	Patient	1729	NHS supply chain FVQ1781
TELA ovitex PRS 10x12cm	Patient	1764	NHS supply chain FXS618
Theatre consumables	Patient	1231.09	UHS preference cards for pelvic exenteration non-routine items, including harmonic scalpel and staple devices
Theatre minutes	Cutting minute	2	Local Finance Department: Includes theatre facilities, recovery, routine associated consumables, anaesthetic drugs, and all non-medical staffing
Consultant per hour	Hour	107	PSSRU 11.3.2<br><br>Assumptions:<br>One consultant anaesthetist will be in theatre at all times.<br><br>Conventional case: one consultant and two trainees will be scrubbed, with supplementary teams as below<br><br>High-complexity case: two consultants and one trainee will be allocated for the duration, with supplementary teams as below.<br><br>If vascular, urology or gynaecology scrubbed assume

			they are present for 30% of the case.<br><br>If plastics present assume they are scrubbed for 40% of the case.<br><br>If orthopaedics, neurospinal, HPB, or UGI present they are scrubbed for 20% of the case
Registrar per hour	Hour	50	PSSRU 11.3.2
**Admission costs**			
Intensive care unit	Bed-day	2479	NHS Reference Costs (weighted average of XC01Z – XC05Z)
High-dependency unit	Bed-day	1645	NHS Reference Costs (XC06Z)
Inpatient routine ward	Bed-day	408.86	NHS Reference Costs – freedom of information request to access 2022 costs, converted to 2023 with Bank of England Inflation Calculator
Standard red cells	Unit	450	NHS Reference Costs (SA44A)
**Investigatations for complications or oncological surveillance**			
CT head	Unit	134	NHS Reference Costs (RD20A)
MRI pelvis	Unit	263	NHS Reference Costs (RD02A)
MRI spine	Unit	231	NHS Reference Costs (RD01A)
MRI head	Unit	231	NHS Reference Costs (RD01A)
CT abdomen and pelvis	Unit	154	NHS Reference Costs (RD24Z)
CT chest, abdomen, and pelvis	Unit	172	NHS Reference Costs (RD26Z)
CT kidneys, ureters, bladder	Unit	134	NHS Reference Costs (RD20A)
CT angiogram	Unit	154	NHS Reference Costs (RD24Z)
CT urogram	Unit	161	NHS Reference Costs (RD22Z)
CT pulmonary angiogram	Unit	147	NHS Reference Costs (RD21A)
US doppler	Unit	106	NHS Reference Costs (RD47Z)
US KUB	Unit	93	NHS Reference Costs (RD40Z)
US abdomen	Unit	93	NHS Reference Costs (RD40Z)
Magnetic resonance cholangiopancreatogram	Unit	231	NHS Reference Costs (RD01A)
US neck	Unit	93	NHS Reference Costs (RD40Z)
US pelvis	Unit	93	NHS Reference Costs (RD40Z)
US soft tissue	Unit	93	NHS Reference Costs (RD40Z)
Loopogram	Unit	284	NHS Reference Costs (RD31Z)
Renogram	Unit	460	NHS Reference Costs (RN25A)
Gastroscopy	Unit	654	NHS Reference Costs (FE22Z) – Day case

Colonoscopy	Unit	847	NHS Reference Costs (FE32Z) – Outpatient
**Costs for re-interventions**			
Nephrostomy	Unit	523	NHS Reference Costs (YL11Z) – Outpatient
Cholecystostomy	Unit	782	NHS Reference Costs (YG06Z) - Outpatient
CT guided pelvic drain	Unit	958	Local Finance Department
US guided pelvic drain	Unit	222	Local Finance Department
CT guided abdominal drain	Unit	958	Local Finance Department
US guided abdominal drain	Unit	222	Local Finance Department
US guided soft tissue drain	Unit	222	Local Finance Department
Fluroscopic drain in IR (pelvis)	Unit	341	Local Finance Department
Inferior vena cava filter	Unit	562	NHS Reference Costs (YR22C)
Flexible cystoscopy	Unit	249	NHS Reference Costs (LB72A) - Outpatient
Percutaneous attention to ureteric stent	Unit	311	NHS Reference Costs (YL12Z) - Outpatient
Emergency operating theatre (for complications)	Cutting Minute	23	Local Finance Department as above, note medical staffing assumed to be on call
**Adjuvant treatment costs***			
Simple Parenteral Chemotherapy	Unit	217	NHS Reference Costs (SB12Z) - Outpatient
Deliver Chemotherapy inclduing Prolonged Infusional Treatment	Unit	361	NHS Reference Costgs (SB14Z) - Outpatient
Apalutamide	240mg x28 tables	2,735	BNF
Bevacizumab	500mg x1 Avastin vial<br>100mg x1 Avastin vial	242.66<br>924.40	BNF
Cabroplatin	150mg x1 vial<br>600mg x1 vial	20.22<br>71.44	eMIT DHE001<br>eMIT DHA162
Capecitabine	500mg x120 tablets	22.51	eMIT DHA225
Cetuximab	500mg x1 vial	890.50	BNF
Cisplatin	100mg x1 vial	29.27	eMIT DHA010
Fluorouracil	500mg x 10 vials	63.76	eMIT DHA026
Folinic acid	350mg x10 vials	61.15	eMIT DHA161
Gemcitabine	2000mg x 1 infusion bag	45.96	eMIT DHB248
Irinotecan	360mg x1 vial	70.94	eMIT DLK054
Niraparib	100mg x56 tablets	6,750	BNF
Oxaliplatin	200mg x1 vial	14.30	eMIT DHC072
Paclitaxel	300mg x1 vial	24.43	eMIT DHA210
Delivery of a single fraction of intracavitary radiotherapy	Unit	1,165	NHS Reference Costs (SC26Z) - Outpatient

Table S1 – Unit costs for health resources used by item, unit, cost-assignation, and source. Note that doses of adjuvant treatment were derived from Southampton systemic chemotherapy protocols assuming $2m^2$ body surface area and 70kg weight dosing was used for all patients. NHS = National Health Service, eMIT = Drugs and Pharmaceutical electronic market information tool, BNF = British National Formulary, UHS = University Hospital Southampton, US = ultrasound, CT = computed tomography, MRI = magnetic resonance imaging, UGI = upper gastrointestinal, HPB = hepato-pancreato-biliary.
