# Supplementary material for: A Prospective Observational Cohort Study Comparing High-Complexity Against Conventional Pelvic Exenteration Surgery
Source: Cancers (Basel). 2025 Jan 1;17(1):111. doi: 10.3390/cancers17010111 (PMC11719841; doi:10.3390/cancers17010111)
Supplement: Supplementary file 1 [file cancers-17-00111-s001.zip › Table S2 - Detailed PROM analysis.pdf]

PROM Score	Months post-PE *(n)*							
	Baseline – 0		3		6		12	
**Decision Regret Scale, medians (IQR), p-value**								
C-PE	12.5 (8.75)	0.40	2.5 (10)	0.47	0 (8.75)	0.17	2.5 (5)	0.90
HC-PE	15 (23.8)		5 (10)		5 (13.8)		5 (10)	
**EQ5D-5L, medians (IQR), p-value**								
5L-Utility score								
C-PE	0.91 (0.05)	0.14	0.75 (0.15)	0.68	0.92 (0.16)	0.16	0.86 (0.14)	0.51
HC-PE	0.86 (0.12)		0.78 (0.26)		0.81 (0.26)		0.83 (0.23)	
Visual analogue score								
C-PE	72.5 (13.8)	0.20	75.0 (15)	0.56	78.5 (10)	0.14	85.0 (15)	0.31
HC-PE	80.0 (15)		70.0 (20)		75.0 (15)		77.5 (30)	
Mobility								
C-PE	1.0 (0)	0.94	1.5 (1)	0.29	1.0 (0.75)	**0.04**	1.0 (1)	0.38
HC-PE	1.0 (0)		2.0 (2)		2.0 (2)		1.0 (2)	
Self-care								
C-PE	1.0 (0)	0.45	1.5 (1)	0.58	1.0 (0)	0.37	1.5 (1)	0.65
HC-PE	1.0 (0)		1.0 (1)		1.0 (1)		1.0 (1)	
Usual activities								
C-PE	1.0 (0)	0.72	2.0 (1)	0.84	1.5 (1)	0.30	2.0 (1)	0.25
HC-PE	1.0 (0.75)		2.0 (1.75)		2.0 (2)		2.0 (2)	
Pain / discomfort								
C-PE	1.5 (1)	0.29	2.0 (1)	0.12	1.0 (1)	**0.049**	1.0 (0.75)	0.052
HC-PE	2.0 (2)		2.0 (1)		2.0 (2)		2.0 (2)	
Anxiety / depression								
C-PE	1.5 (1)	0.15	2.0 (1)	0.95	1.0 (0)	0.36	1.0 (1)	0.35
HC-PE	2.0 (1)		1.5 (1)		1.0 (1)		2.0 (1.25)	

Table S2 - Detailed patient-reported outcome measure scoring comparisons. Note that all 10 patients in the conventional pelvic exenteration (C-PE) group completed 12-month follow-up. There were 45, 42, and 36 PROMs returned at 3-months, 6-months, and 12-months respectively in the high-complexity PE (HC-PE) group. Analysis was as per original assigned groups. IQR = interquartile range.
