# Supplementary material for: A Prospective Observational Cohort Study Comparing High-Complexity Against Conventional Pelvic Exenteration Surgery
Source: Cancers (Basel). 2025 Jan 1;17(1):111. doi: 10.3390/cancers17010111 (PMC11719841; doi:10.3390/cancers17010111)
Supplement: Supplementary file 1 [file cancers-17-00111-s001.zip › Table S3 - Health economic sub-analyses.pdf]

**A) Major vascular reconstruction cases**	**No NEV Resection**	**NEV Resection**	**P-value**
**Sample size,** *n (%)*	290 (92)	24 (8)	
Overall costs (£), *mean (SD)*	43,135 (22,484)	54,909 (26,411)	0.04

**B) Major excisions of the pelvic bone**	**No bone resection**	**Major bone resection**	**P-value**
**Sample size,** *n (%)*	252 (80)	62 (20)	
Overall costs (£), *mean (SD)*	38,787 (20,074)	65,366 (21,775)	<0.001

**C) All infralevator vs all supralevator PE**	**Supralevator PE**	**Infralevator PE**	**P-value**
**Sample size,** *n (%)*	191 (61)	123 (39)	
Overall costs (£), *mean (SD)*	38,787 (20,074)	65,366 (21,775)	<0.001

**D) Sub-analysis of levator resection by complexity**	**Sample size,** *n (%)*	**Overall costs (£),** *mean (SD)*	**P-value**
Conventional supralevator PE	37 (12)	31,499 (14,363)	
High-complexity supralevator PE	154 (49)	36,262 (18,567)	
Conventional infralevator PE	26 (8)	45,485 (12,452)	<0.001
High-complexity infralevator PE	97 (31)	60,769 (24,756)	

Table S3 – Cost-effectiveness sub-analyses. A) Comparison of particular components of interest in increasing levels of cost. Non-expendable vessel (NEV) resection, defined as the common (E4) or external iliac vessels (SV4) as per UK Pelvic Exenteration Network Lexicon (UKPEN). B) Major bone, defined as sacrum (≥P2), pubic bone (A4), ischial spine (PM2), or iliac crest (PM3). C) Infralevator PE (PM1 or PM2) vs supralevator PE (PM0 or PM3). D) Sub-analysis of levator resection divided further by complexity, ANOVA derived p-value, note on post-hoc testing only conventional vs high-complexity supralevator PE (p=1.00), and high-complexity supralevator PE vs conventional infralevator PE (p=0.18) were not significant.
