# Supplementary material for: A Prospective Observational Cohort Study Comparing High-Complexity Against Conventional Pelvic Exenteration Surgery
Source: Cancers (Basel). 2025 Jan 1;17(1):111. doi: 10.3390/cancers17010111 (PMC11719841; doi:10.3390/cancers17010111)
Supplement: Supplementary file 1 [file cancers-17-00111-s001.zip › Table S4 - Health economic sensitivity analysis.pdf]

	Conventional PE (£)		High-complexity PE (£)	
Scenario altered	Cost per QALY	No PE - ICER	Cost per QALY	No PE - ICER
**Base case**	7,414	2,446	10,077	5,061
**No exenteration**				
+50% Survival		1,391		4,769
-50% Survival		3,081		5,542
+50% Utility		2,901		6,252
-50% Cost		6,093		9,488
**Conventional PE**				
-20% Survival	7,919	2,607		
-20% Utility	9,268	3,317		
+20% peri-operative cost	8,649	4,068		
+50% post-discharge cost	8,034	3,260		
**High-complexity PE**				
-20% Survival			10,796	5,503
-20% Utility			12,596	6,992
+20% peri-operative cost			11,826	7,477
+50% post-discharge cost			10,741	5,978

Table S4 – Scenario-testing for deterministic sensitivity analysis, variables of the Markov model are varied to unfavour pelvic exenteration (PE) and reduce its cost-utility. To capture the increased uncertainty of no-PE and post-discharge costs, these are increased to 50%. Post-discharge costs encompassed costs for complications, adjuvant treatments, and surveillance imaging. QALY = quality-adjusted life-year, ICER = incremental cost-effectiveness ratio.
